# Combining PD-1/PD-L1 blockade with type I interferon in cancer therapy

**DOI:** 10.3389/fimmu.2023.1249330

**Published:** 2023-08-24

**Authors:** Ali Razaghi, Mickaël Durand-Dubief, Nele Brusselaers, Mikael Björnstedt

**Affiliations:** ^1^ Department of Laboratory Medicine, Division of Pathology, Karolinska Institutet, Karolinska University Hospital, Stockholm, Sweden; ^2^ Discovery & Front-End Innovation, Lesaffre Institute of Science & Technology, Lesaffre International, Marcq-en-Baroeul, France; ^3^ Global Health Institute, Antwerp University, Antwerp, Belgium; ^4^ Centre for Translational Microbiome Research (CTMR), Department of Microbiology, Tumor and Cell Biology, Karolinska Institute, Karolinska Hospital, Stockholm, Sweden; ^5^ Department of Head and Skin, Ghent University, Ghent, Belgium

**Keywords:** cancer, interferon, immunotherapy, PD-1, PD-L1, interferon a (IFNa)

## Abstract

PD-1 and PD-L1 are crucial regulators of immunity expressed on the surface of T cells and tumour cells, respectively. Cancer cells frequently use PD-1/PD-L1 to evade immune detection; hence, blocking them exposes tumours to be attacked by activated T cells. The synergy of PD-1/PD-L1 blockade with type I interferon (IFN) can improve cancer treatment efficacy. Type I IFN activates immune cells boosts antigen presentation and controls proliferation. In addition, type I IFN increases tumour cell sensitivity to the blockade. Combining the two therapies increases tumoral T cell infiltration and activation within tumours, and stimulate the generation of memory T cells, leading to prolonged patient survival. However, limitations include heterogeneous responses, the need for biomarkers to predict and monitor outcomes, and adverse effects and toxicity. Although treatment resistance remains an obstacle, the combined therapeutic efficacy of IFNα/β and PD-1/PD-L1 blockade demonstrated considerable benefits across a spectrum of cancer types, notably in melanoma. Overall, the phases I and II clinical trials have demonstrated safety and efficiency. In future, further investigations in clinical trials phases III and IV are essential to compare this combinatorial treatment with standard treatment and assess long-term side effects in patients.

## Introduction

Cancer cells frequently exploit the Programmed Cell Death 1 (PD-1) signalling pathway to evade immune surveillance. Antibodies (Abs) that target PD-1 and its ligand PD-L1 can rescue exhausted T cells and revive immune responses against cancer cells. To date, anti-PD-1 Abs (camrelizumab cemiplimab, dostarlimab, nivolumab, pembrolizumab, prolgolimab, sintilimab, tislelizumab, toripalimab and zimberelimab) and anti-PD-L1 Abs (atezolizumab, avelumab, and durvalumab) have been approved for treating Hodgkin lymphoma, urothelial, hepatocellular, oesophagal, renal cell carcinomas, malignant pleural mesothelioma, head and neck, colorectal, skin, non-small cell lung, gastric, gastroesophageal junction, bladder, cervical and endometrial cancers (1).

Effective control of tumours by PD-1/PD-L1 therapy is associated with a higher level of tumour-infiltrating lymphocytes (TILs) (2). However, anti-PD1/PDL1 therapy benefits only a subset of patients. (3), *i.e.* PD-1/PD-L1 blockade therapy may not be sufficient to (re-) activate tumour-specific T lymphocytes (even in the presence of TILs) leading to intrinsic resistance (4). Furthermore, after initial responses, a large group of responders may develop acquired resistance. Major histocompatibility complex (MHC) dysfunction has been identified as one of the main resistance mechanisms to PD-1/PD-L1 therapy because antigen presentation in the tumour microenvironment (TME) is primarily accomplished via the MHC class I pathway. Consequently, tumours can evade T cell killing through inactivating the MHC class I complex. In other words, the presence of putative tumour rejection antigens provided by dendritic cells (DCs) to cross-priming CD8^+^ T lymphocytes results in less anticancer activity and inefficacy of PD-1/PD-L1 blockade (3).

IFN type I (IFNα, IFNβ, IFNω, IFNε, and IFNк) has multiple anti-tumour activities, such as direct tumour cell killing and the stimulation of immune cells including DCs and CD8^+^ T cells (5–7). To date, IFNα/β have been approved for the clinical treatment of multiple malignancies (*e.g.*, Kaposi’s sarcoma, melanoma and renal cell carcinoma). However, monotherapy of recombinant IFNα/β is not well tolerated when administered systemically (causing a range of side effects in humans including fatigue, fever, muscle aches, depression, and liver damage) (8). To overcome monotherapy challenges with either IFNα/β or PD-1/PD-L1 blockade therapies; a combination of these two immunotherapies has been proposed to circumvent the resistance to PD-1/PD-L1 therapies and improve patient outcomes. For example, in patients with immune cell-poor melanomas, stimulating type I IFN puts forwards a rational approach to boost the therapeutic benefits of PD-1/PD-L1 inhibition (9).

This review gathered and systematically analyses the latest developments in combining PD-1/PD-L1 blockade with type I IFN application in preclinical and clinical stages to shed light on the current status and future research.

## Type I IFN reinvigorates immune cells: mechanism of action

Inducing efficient tumour-specific cytotoxic T-cell responses is one of the objectives of anticancer therapies. DC can stimulate cross-priming with CD8^+^ T cells, known as cross-presentation by which antigen-presenting cells present tumour-associated antigens on their MHC class-I molecule (5). Type I IFN stands among the most potent activators for DC-induced cross-priming. Preclinical evidence suggests that type I IFN-stimulated cross-priming of DC against tumour-associated antigen is crucial for cancer immunosurveillance and can be used to effectively increase anti-tumour CD8^+^ T-cell responses (5). Mechanistically, type I IFNs promote the production of IP-10/CXCL10, a chemokine for attracting effector T cells to the TME. Type I IFNs also induces overexpression of MHC class I on tumour cells enhancing the effector response of anti-tumour CD8^+^ T cell in the TME (10). However, Type I IFN expression is limited or repressed within the TME. For instance, reduced cGAS-STING pathway signalling in certain tumour cells and the enhanced degradation of DNA and antigen within the TME can inhibit innate sensing and type I IFN production (11). Thereby, the intravenous application of IFNα/β might be an option to overcome limited expression in the TME. For example, in melanoma patients, peritumoral injection of IFNβ is known for recruiting CD8^+^ cytotoxic T cells, into the TME. This finding may elucidate the therapeutic advantages of IFNβ in melanoma treatment (12). Furthermore, IFNβ favours a shift in the phenotypes of tumour-associated macrophages from M_2_ to M_1_ phenotype, thereby reducing the proportion of Tregs among TILs within the TME (13). In murine melanoma, IFNβ increases the effects of anti-PD-1 Abs against melanoma by preferentially drawing effector cells, rather than Tregs, to tumour sites (13).

## Current status

**Figure 1:**
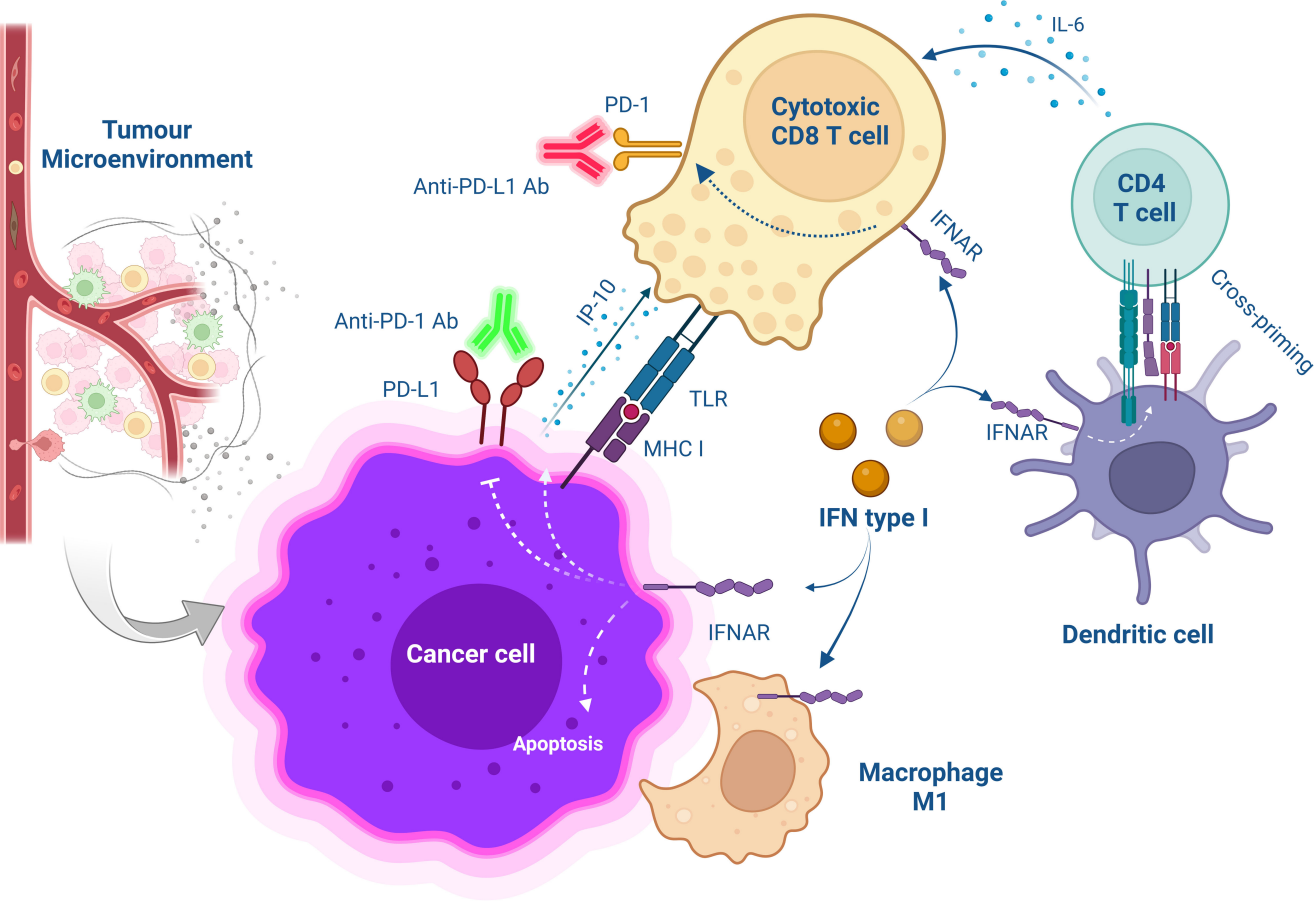
Schematic overview of cellular interaction after combinational treatment with type I IFN and PD-1/PD-L1 blockade. The treatment with anti-PD-1/PD-L1 Abs saves CD8^+^ T cells from exhaustion while targeting IFNα to the tumour microenvironment promotes the release of IP-10 from antigen-positive tumour cells increases T cell infiltration and improves CD4^+^ T cell function for anti-tumour immunity. Furthermore, MHC class I are upregulated on cancer cells increasing antitumor CD8^+^ T cell response. Also, IFNα activates DC-induced cross-priming by releasing IL-6.

Combining PD-1/PD-L1 blockade with type I IFN presents potential benefits, including the activation of innate and adaptive immune cells, enhancement of antigen presentation on DCs, induction of PD-L1 expression on tumour cells, and sensitising cancer cells to PD-1/PD-L1 blockade (14) (**Figure 1**). Several preclinical and clinical studies have demonstrated that combining PD-1/PD-L1 blockade with IFN shows a therapeutically synergistic effect by increasing intratumoral T cell infiltration and activation, generating memory T cells, and prolonging the survival of both animals and patients (14, 15) (**Tables 1**, **2**).

**Table 1:** Preclinical studies utilizing a combination of PD-1/PD-L1 blockade with type I IFN in cancer therapy.

Summary	Methods	Reference
IFNα induces PD-1 expression in antigen-specific CD8^+^ T cells and on TCR-engaged mouse T cells through the IFN-responsive factor 9.	Murine colon adenocarcinoma (*in vivo*)	(16)
Anti-PD-1-blockade Ab along with poly (I:C) treatment regressed the established mouse melanomas, and increased survival time when compared to poly (I:C) treatment alone (126 ± 16 vs. 97 ± 13 days). But anti–PD-1 Ab alone did not demonstrate any effect. As a result, targeting type I IFNs in conjunction with blocking the PD-L1-PD-1 signalling pathway can be a strategy for extending immune surveillance.	Primary melanomas in Hgf-Cdk4R24C mice imitating human immune cell–poor melanomas (*in vivo*)	(9)
Peritoumorally administered IFNβ increases PD-1 expression on TILs, boosts anti-PD-1 Ab’s anti-tumour immune response, and reduces mRNA expression and Th2-related chemokine production, thereby suppressing Treg recruitment. While the combination treatment with anti-PD-1 Ab increased the therapeutic impact of IFNβ.	B16F10 melanoma in mice (*in vivo*)	(13)
Co-culture of melanoma cells with immune cells increases tumour cell PD-L1 expression. The interferon-related secretome also promotes PD-L1 expression.	Co-culture of B16F10 melanoma cells with bone marrow cells, lymph node cells, or splenocytes (*in vitro*)	(17)
IFNα-derived AcTaferon-targeting melanoma displayed more potent antitumor activity (>1,000-fold) compared to wild-type IFNα. Combined with anti-PD-L1 Ab blockade complete tumour regression was observed without toxicity.	Clec9A+ DCs B16 melanoma, lymphoma, breast carcinoma and against human lymphoma cells in mouse (*in vivo*)	(18)
Treatment of tumours with CD20-targeted AcTaferon (coupled hIFNa2-Q124R9 to PD-L1 Ab targeting mCD20) significantly shrunk tumour growth compared to anti-PD-L1 Ab treatment alone (P < 0.0001). Accordingly, combinational treatment may convert nonresponding tumours into responders.	PD-L1 expression was analysed on B16 (melanoma)-mCD20 cells *in vitro* and *in vivo* (spleen C tumour)	(19)
Local delivery of IFNα restores antigen presentation but upregulates PD-L1 dampening subsequent T-cell activation. To overcome therapy resistance. The fusion protein of IFNα-anti-PD-L1 can create feedforward synergistic effects activating IFNAR signalling in host cells.	293F (HEK293) cell line (*in vitro* and *in vivo*)	(11)
IFNα-2b treatment of DC increased the surface expression of PD-L1, the release of IL-6, whereas decreased IL-12 production. IFNα-2b inhibits DC stimulation by inducing STAT3/p38-mediated expression of PD-L1.	Xenograft of human DCs in mice (*in vivo*)	(20)
IFNβ upregulates the expression of PD-1 on NK cells (5-fold) and increased the expression of PD-L1 on neural progenitor cells (6 to 13-fold). Concurrent treatment of NK- and neural progenitor cells with IFNβ before coculture caused NK cells to release intracellular TRAIL and lowered cytotoxicity.	Patient-derived xenograft nasopharyngeal carcinoma in mice (*in vivo*)	(21)
Anti-PD-L1-mIFNα promotes IP-10 release from antigen-positive tumour cells, increasing T cell infiltration, and improving effector T cell function for anti-tumour immunity. Also, it upregulates MHC class I on tumour cells, increasing the response of the antitumour CD8^+^ T cells. Hence, the anti-PD-L1-mIFNα moiety can be a useful method for reducing tumour resistance to PD-L1 inhibition.	Murine tumour cell lines (LL/2-OVA and MC38-OVA) expressing chicken ovalbumin (*in vitro* and *in vivo*)	(10)
The combination of PD-1 inhibition with pegylated-IFNα had a synergistic effect, increased the efficacy of PD-1 Ab and restored CD8^+^ T cell cytotoxicity. *i.e.*, improved T-cell infiltration and significantly extended mouse survival compared to control or single agent (p<0.01). Pegylated-IFNα induces tumour cells to secrete the chemokine CCL4 and recruits cytotoxic CD8^+^ T cells to infiltrate the TME, consequently overcoming immune responses by increasing PD-1 expression in CD8^+^ T cells via the IFNAR1-JAK1-STAT3 signalling pathway.	Human hepatocellular cells and murine cell lines (Hepa1-6) in mice (*in vivo*)	(14)

**Table 2:** Clinical trials of a combination of PD-1/PD-L1 blockade with type I interferon in cancer therapy.

Summary	Clinical stage	Reference
The combined safe dose was determined to be 3x10^6^ units. The rate of complete tumour response was ~22% (95% CI), higher than nivolumab alone (~9%). Overall, IFNβ does not increase the rate of immune-related adverse events and may even enhance nivolumab’s anti-melanoma effects.	Phase I, IFNβ plus nivolumab in metastatic melanoma (9 patients).	(6)
The combinatorial treatment was well-tolerated and showed promising efficacy in the treatment of melanoma. Additionally, the treatment was found to have a positive impact on the T-cell repertoire enhancing the immune response against melanoma cells.	Phase I, a combination treatment of neoadjuvant ipilimumab (3 mg/kg or 10 mg/kg) and high dose IFNα-2b in patients with advanced melanoma (30 patients).	(22)
The standard dose (2 mg/kg) of pembrolizumab plus pegylated IFNα-2b (1 μg/kg/week) was identified as the maximum tolerated dose. However, a poor tolerability profile and minimal antitumor activity were observed.	Phase Ib, combining pembrolizumab with either pegylated IFMα-2b for the treatment of advanced melanoma or renal cell carcinoma (17 patients)	(23)
The combinational treatment pembrolizumab (2 mg/kg) and pegylated-IFN (3 μg/kg) per week showed to be an active and safe option for patients with the objective response rate (ORR) ~23%.	Phase Ib/II, a combination of pembrolizumab and pegylated-IFNα-2b in metastatic melanoma (PD-1-naïve melanoma) (26 patients)	(24)
The combinatorial treatment was largely well-tolerated, with side effects being mild or moderate. The overall response rate was 61%, with 14 patients having a complete or partial response. The pathologic response rate was 78%, indicating significant tumour shrinkage with a median disease-free survival (~22 months) and a 2-year overall survival rate (74%). Overall, the treatment was considered efficient with hypophosphatemia and fatigue as side effects.	Phase Ib/II, neoadjuvant pembrolizumab (200 mg intravenously every 3 weeks) and high-dose IFNα-2b (intravenously 20 MU/m^2^/day, 5 days/week for 4 weeks, followed subcutaneously 10 MU/m^2^/day, 3 days/week for 2 weeks) were given to resectable stage III melanoma in two cycles before surgery (30 patients).	(25)
Prior pegylated IFNα-2b therapy improved the efficacy of subsequent adjuvant pembrolizumab and increased recurrence-free survival (median 8.5 vs. 4.5 months).	Clinical retrospective analysis for melanoma (25 patients)	(26)

To date, the phases I and II clinical trials (~130 patients in total) determined the optimal dose and showed that the combinatorial treatment of PD-1/PD-L1 blockade with IFNα/β is safe and efficient for particularly melanoma and to some extent for renal carcinoma patients (**Table 2**). Yet, further clinical phases III and IV trials are required to compare this combinatorial treatment with standard treatment and also to assess the side effects in the long term in patients. Later, other cancer types can also be evaluated in Phase III clinical trials for the effectiveness of this combinational treatment in larger populations.

## Limitations and future prospects

Despite the determination of optimal dose and adverse effects of the combined type I IFN and PD-1/PD-L1 blockade therapy in phase I and II clinical trials (**Table 2**), challenges and limitations still include the need for biomarkers to predict and monitor treatment outcomes, understanding the tumour response across different cancer types and patients and more investigation on the optimal dose, timing and the potential toxicity and adverse events in later stages clinical trials (14). To address these limitations, the following suggestions might be considered.

Since the 1990s, clinical cancer research using DC-based vaccination has demonstrated validated safety and feasibility. However, DC vaccinations are still a new strategy for acute myeloid leukaemia (5-year overall survival rate >30%). Limitations include weak immune responses, time-consuming processes and high costs (27). Therefore, DC vaccination combined with immune checkpoint blockades such as PD-1 Abs may enhance DC-mediated activation of NK and T cells and prevent Treg stimulation (27, 28). In 2018, a phase II clinical trial indicated that activated autologous DC paired with PD-1 blockade (pembrolizumab) has a positive effect in patients with advanced solid tumours (ORR ~22%). This approach enabled the release of the brake on active T cells by inhibiting the PD-1/PD-L1 checkpoint leading to increased immune responsiveness and tumour elimination (29). In addition, both preclinical and clinical studies have indicated that combining DC vaccination and IFNα can reinvigorate the immune response against cancer cells, leading to improved outcomes (30). Thus, a combination of PD-1/PD-L1 checkpoint inhibitors with DC vaccines (reinvigorated by IFNα) might be a viable strategy to stimulate the patient’s immune system against the tumour in advance (30).

In contrast to general positive perception towards combined type I IFN and PD-1/PD-L1 blockade, one study debated that sustained type I IFN signalling may contribute to resistance to PD-1/PD-L1 blockade, by inducing nitric oxide synthase 2 (NOS_2_) expression in tumour and DCs, leading to T cell dysfunction and Treg accumulation (31). Also, resistance to anti-PD-1 monotherapy in melanoma patients was correlated with the induction of a type I IFN signature (31). Hence, this controversy may require more investigation in future.

Overall, the prospective effectiveness of a combined PD-1/PD-L1 blockade with type I IFN in cancer treatment depends on numerous factors including the type and dose of IFNα/β, the timing and duration of treatment, the tumour type and stage of cancer, the genetic and epigenetic alterations of tumour cells, the immune status of patients, and the interactions with other therapeutic interventions. Additional preclinical and clinical studies are crucial to elucidate the optimal conditions and underlying mechanisms of this combination strategy.

## Conclusion

PD-1 serves as a receptor on T cells whereas PD-L1 is a ligand present in cancer cells or antigen-presenting cells, the latter of which exposes antigens to T cells. The binding of PD-1 and PD-L1 sends a signal that reduces the activity and survival of T cells. By blocking PD-1/PD-L1 interaction, the immune response against cancer cells can be enhanced.

Type I IFN represents a family of cytokines that exhibit dual roles in cancer immunity, manifesting both beneficial and detrimental effects. On one hand, type I IFN can increase the expression of antigens and co-stimulatory molecules on cancer cells and antigen-presenting cells, thereby increasing their visibility to T cells. Type I IFN can also stimulate the production and function of T cells and other immune cells, such as NK cells and DCs. On the other hand, type I IFN can induce the expression of PD-L1 inhibiting T cell function. Type I IFN also activates the NOS_2_ enzyme, which through the production of nitric oxide, can suppress T cell activity and promote the accumulation of Treg, subsequently followed by suppression of other immune cells.

Overall, the phases I and II clinical trials have demonstrated safety and efficiency, particularly for melanoma patients and to some extent for renal carcinoma patients. Further investigations in clinical trials phases III and IV are still needed to compare this combinatorial treatment with standard treatment and assess long-term side effects in patients. Later, other cancer types can also be evaluated in Phase III clinical trials for the effectiveness of this combinational treatment in larger populations.

## Author contributions

AR: collecting information, writing and editing the manuscript and visualization. MD-D and NB: reviewing and editing the manuscript. MB: editing and funding. All authors contributed to the article and approved the submitted version.

## References

[1] Yi M Zheng X Niu M Zhu S Ge H Wu K. Combination strategies with PD-1/PD-L1 blockade: current advances and future directions. Mol Cancer (2022) 21:28. doi: 10.1186/s12943-021-01489-2 35062949 PMC8780712

[2] Tumeh PC Harview CL Yearley JH Shintaku IP Taylor EJ Robert L. PD-1 blockade induces responses by inhibiting adaptive immune resistance. Nature (2014) 515:568–71. doi: 10.1038/nature13954 PMC4246418 25428505

[3] Lei Q Wang D Sun K Wang L Zhang Y. Resistance mechanisms of anti-PD1/PDL1 therapy in solid tumors. Front Cell Dev Biol (2020) 8:672. doi: 10.3389/fcell.2020.00672 32793604 PMC7385189

[4] Huang AC Postow MA Orlowski RJ Mick R Bengsch B Manne S. T-cell invigoration to tumour burden ratio associated with anti-PD-1 response. Nature (2017) 545:60–5. doi: 10.1038/nature22079 PMC5554367 28397821

[5] Schiavoni G Mattei F Gabriele L. Type I interferons as stimulators of DC-mediated cross-priming: impact on anti-tumor response. Front Immunol (2013) 4:483. doi: 10.3389/fimmu.2013.00483 24400008 PMC3872318

[6] Fujimura T Hidaka T Kambayashi Y Furudate S Kakizaki A Tono H. Phase I study of nivolumab combined with IFN-beta for patients with advanced melanoma. Oncotarget (2017) 8:71181–7. doi: 10.18632/oncotarget.17090 PMC5642629 29050354

[7] Shi W Yao X Fu Y Wang Y. Interferon-alpha and its effects on cancer cell apoptosis. Oncol Lett (2022) 24:235. doi: 10.3892/ol.2022.13355 35720476 PMC9185151

[8] Razaghi A Brusselaers N Bjornstedt M Durand-Dubief M. Copy number alteration of the interferon gene cluster in cancer: Individual patient data meta-analysis prospects to personalized immunotherapy. Neoplasia (2021) 23:1059–68. doi: 10.1016/j.neo.2021.08.004 PMC8458777 34555656

[9] Bald T Landsberg J Lopez-Ramos D Renn M Glodde N Jansen P. Immune cell-poor melanomas benefit from PD-1 blockade after targeted type I IFN activation. Cancer Discovery (2014) 4:674–87. doi: 10.1158/2159-8290.CD-13-0458 24589924

[10] Guo J Xiao Y Iyer R Lu X Lake M Ladror U. Empowering therapeutic antibodies with IFN-alpha for cancer immunotherapy. PloS One (2019) 14:e0219829. doi: 10.1371/journal.pone.0219829 31393905 PMC6687177

[11] Liang Y Tang H Guo J Qiu X Yang Z Ren Z. Targeting IFNalpha to tumor by anti-PD-L1 creates feedforward antitumor responses to overcome checkpoint blockade resistance. Nat Commun (2018) 9:4586. doi: 10.1038/s41467-018-06890-y 30389912 PMC6214895

[12] Fujimura T Okuyama R Ohtani T Ito Y Haga T Hashimoto A. Perilesional treatment of metastatic melanoma with interferon-beta. Clin Exp Dermatol (2009) 34:793–9. doi: 10.1111/j.1365-2230.2009.03207.x 19438554

[13] Kakizaki A Fujimura T Furudate S Kambayashi Y Yamauchi T Yagita H. Immunomodulatory effect of peritumorally administered interferon-beta on melanoma through tumor-associated macrophages. Oncoimmunology (2015) 4:e1047584. doi: 10.1080/2162402X.2015.1047584 26451326 PMC4589056

[14] Zhu Y Chen M Xu D Li TE Zhang Z Li JH. The combination of PD-1 blockade with interferon-alpha has a synergistic effect on hepatocellular carcinoma. Cell Mol Immunol (2022) 19:726–37. doi: 10.1038/s41423-022-00848-3 PMC9151669 35459855

[15] Burrack AL Spartz EJ Raynor JF Wang I Olson M Stromnes IM. Combination PD-1 and PD-L1 blockade promotes durable neoantigen-specific T cell-mediated immunity in pancreatic ductal adenocarcinoma. Cell Rep (2019) 28:2140–2155 e2146. doi: 10.1016/j.celrep.2019.07.059 31433988 PMC7975822

[16] Terawaki S Chikuma S Shibayama S Hayashi T Yoshida T Okazaki T. IFN-alpha directly promotes programmed cell death-1 transcription and limits the duration of T cell-mediated immunity. J Immunol (2011) 186:2772–9. doi: 10.4049/jimmunol.1003208 21263073

[17] Yang YQ Dong WJ Yin XF Xu YN Yang Y Wang JJ. Interferon-related secretome from direct interaction between immune cells and tumor cells is required for upregulation of PD-L1 in tumor cells. Protein Cell (2016) 7:538–43. doi: 10.1007/s13238-016-0281-6 PMC4930771 27295261

[18] Cauwels A Van Lint S Paul F Garcin G De Koker S Van Parys A. Delivering type I interferon to dendritic cells empowers tumor eradication and immune combination treatments. Cancer Res (2018) 78:463–74. doi: 10.1158/0008-5472.CAN-17-1980 29187401

[19] Cauwels A Van Lint S Garcin G Bultinck J Paul F Gerlo S. A safe and highly efficient tumor-targeted type I interferon immunotherapy depends on the tumor microenvironment. Oncoimmunology (2018) 7:e1398876. doi: 10.1080/2162402X.2017.1398876 29399401 PMC5790344

[20] Bazhin AV Von Ahn K Fritz J Werner J Karakhanova S. Interferon-alpha up-regulates the expression of PD-L1 molecules on immune cells through STAT3 and p38 signaling. Front Immunol (2018) 9:2129. doi: 10.3389/fimmu.2018.02129 30356906 PMC6190899

[21] Makowska A Braunschweig T Denecke B Shen L Baloche V Busson P. Interferon beta and anti-PD-1/PD-L1 checkpoint blockade cooperate in NK cell-mediated killing of nasopharyngeal carcinoma cells. Transl Oncol (2019) 12:1237–56. doi: 10.1016/j.tranon.2019.04.017 PMC6617170 31295651

[22] Tarhini A Lin Y Lin H Rahman Z Vallabhaneni P Mendiratta P. Neoadjuvant ipilimumab (3 mg/kg or 10 mg/kg) and high dose IFN-α2b in locally/regionally advanced melanoma: safety, efficacy and impact on T-cell repertoire. J Immunother Cancer (2018) 6:112. doi: 10.1186/s40425-018-0428-5 30352626 PMC6199801

[23] Atkins MB Hodi FS Thompson JA Mcdermott DF Hwu WJ Lawrence DP. Pembrolizumab plus pegylated interferon alfa-2b or ipilimumab for advanced melanoma or renal cell carcinoma: dose-finding results from the phase Ib KEYNOTE-029 study. Clin Cancer Res (2018) 24:1805–15. doi: 10.1158/1078-0432.CCR-17-3436 29358500

[24] Davar D Wang H Chauvin JM Pagliano O Fourcade JJ Ka M. Phase Ib/II study of pembrolizumab and pegylated-interferon Alfa-2b in advanced melanoma. J Clin Oncol (2018) 36:JCO1800632. doi: 10.1200/JCO.18.00632 30359157 PMC6286160

[25] Najjar YG Mccurry D Lin H Lin Y Zang Y Davar D. Neoadjuvant pembrolizumab and high-dose IFNalpha-2b in resectable regionally advanced melanoma. Clin Cancer Res (2021) 27:4195–204. doi: 10.1158/1078-0432.CCR-20-4301 PMC8338751 33753453

[26] Jia DD Niu Y Zhu H Wang S Ma T Li T. Prior therapy with pegylated-interferon Alfa-2b improves the efficacy of adjuvant pembrolizumab in resectable advanced melanoma. Front Oncol (2021) 11:675873. doi: 10.3389/fonc.2021.675873 34221994 PMC8243982

[27] Yu J Sun H Cao W Song Y Jiang Z. Research progress on dendritic cell vaccines in cancer immunotherapy. Exp Hematol Oncol (2022) 11:3. doi: 10.1186/s40164-022-00257-2 35074008 PMC8784280

[28] Versteven M Van Den Bergh JMJ Marcq E Smits ELJ Van Tendeloo VFI Hobo W. Dendritic cells and programmed death-1 blockade: A joint venture to combat cancer. Front Immunol (2018) 9:. doi: 10.3389/fimmu.2018.00394 PMC5863527 29599770

[29] Chen CL Pan QZ Weng DS Xie CM Zhao JJ Chen MS. Safety and activity of PD-1 blockade-activated DC-CIK cells in patients with advanced solid tumors. Oncoimmunology (2018) 7:e1417721. doi: 10.1080/2162402X.2017.1417721 29632736 PMC5889206

[30] Lapenta C Gabriele L Santini SM. IFN-alpha-mediated differentiation of dendritic cells for cancer immunotherapy: advances and perspectives. Vaccines (2020) 8(4):617. doi: 10.3390/vaccines8040617 PMC7711454 33086492

[31] Jacquelot N Yamazaki T Roberti MP Duong CPM Andrews MC Verlingue L. Sustained Type I interferon signaling as a mechanism of resistance to PD-1 blockade. Cell Res (2019) 29:846–61. doi: 10.1038/s41422-019-0224-x PMC6796942 31481761

